# Neurogenic thoracic outlet syndrome: A case report and review of the literature

**DOI:** 10.4103/0973-6042.70817

**Published:** 2010

**Authors:** André P Boezaart, Allison Haller, Sarah Laduzenski, Veerandra B. Koyyalamudi, Barys Ihnatsenka, Thomas Wright

**Affiliations:** 1 Department of Anesthesiology, Division of Acute Pain Medicine and Regional Anesthesia, University of Florida, College of Medicine, Gainesville, Florida, United States of American Society of Anesthesiologists; 2 Department Orthopaedic Surgery and Rehabilitation, University of Florida, College of Medicine, Gainesville, Florida, United States of American Society of Anesthesiologists

**Keywords:** Acute pain, botox, neurogenic thoracic outlet syndrome, shoulder pain, thoracic outlet syndrome

## Abstract

Neurogenic thoracic outlet syndrome (NTOS) is an oft-overlooked and obscure cause of shoulder pain, which regularly presents to the office of shoulder surgeons and pain specialist. With this paper we present an otherwise healthy young female patient with typical NTOS. She first received repeated conservative treatments with 60 units of botulinium toxin injected into the anterior scalene muscle at three-month intervals, which providing excellent results of symptom-free periods. Later a trans-axillary first rib resection provided semi-permanent relief. The patient was followed for 10 years after which time the symptoms reappeared. We review the literature and elaborate on the anatomy, sonoanatomy, etiology and characteristics, symptoms, diagnostic criteria and treatment modalities of NTOS. Patients with NTOS often get operated upon – even if just a diagnostic arthroscopy, and an interscalene or other brachial plexus block may be performed. This might put the patient in jeopardy of permanent nerve injury, and the purpose of this review is to minimize or prevent this.

## INTRODUCTION

Neurogenic thoracic outlet syndrome (NTOS) is an oft-overlooked and obscure cause of shoulder pain that regularly presents to the office of shoulder surgeons and pain specialists. We present a typical case of NTOS, and review the anatomy, etiology, characteristics, symptoms, diagnostic criteria, and its treatment modalities. Patients with NTOS usually undergo surgery, if only for a diagnostic arthroscopy, in which case an interscalene or other brachial plexus block may be performed, which may place the patient in jeopardy of permanent nerve injury. The purpose of this article is to, with the aid of a typical case presentation, review the literature on the anatomical, diagnostic, and therapeutic considerations involved with NTOS.

## CASE REPORT

A 19-year-old, slender, Caucasian female patient, who was otherwise healthy, presented with right-sided periscapular and shoulder pain. The nature of the pain was described as a “lame,” nagging type of pain that increased with exercise, and persisted after cessation of activities. Moreover, it was often present at rest. The pain was increased by continuous overhead activity, such as swimming and doing her hair, and downward traction, such as carrying heavy objects.

On examination, the patient was found to be in excellent physical condition, and her neck could be described as slender and “ballerina-like.” Assessment of range of motion in her right shoulder proved negative for signs and symptoms of subacromial or glenohumeral pathology, but the overhead fatigue test (upper arms abducted to 90° and shoulders externally rotated to 90°, while the grip in both hands were squeezed and relaxed) revealed that her right arm developed pain and fatigued, while the left arm did not. The right-sided brachial plexus was very tender upon light palpation compared to the left side, and lateral flexion of the neck aggravated the pain.

The diagnosis of NTOS was considered, and, with the patient lightly sedated with midazolam, and using nerve stimulation (Stimuplex, B Braun, Bethlehem, PA, USA), the brachial plexus and phrenic nerves were identified by eliciting a biceps muscle motor response for the former and a diaphragm motor response for the latter. The anterior scalene muscle was identified between these two nerve landmarks by a negative response to neurostimulation set at an output of 2 mA, a frequency of 2 Hz and a pulse width of 0.3 ms. Five milliliters (mL) of 2% ropivacaine injected into the anterior scalene muscle produced immediate relief of the shoulder and periscapular pain, with no signs of brachial plexus blockade. One hundred units of botulinum toxin were dissolved in 1 mL saline, and 60 units (1 unit/kg) of the toxin were injected into the anterior scalene muscle using the technique described above.

Following these procedures, the patient was entirely free of symptoms, and actively participated in sports, including swimming and tennis, for 3 months until the symptoms returned. The procedure was repeated three more times at approximately 3-month intervals, each time providing the patient with a symptom-free period of 3 months. After the fourth botulinum toxin injection, she was referred to a thoracic surgeon who performed a transaxillary resection of the first rib. After this surgery, the symptoms did not return. The patient was followed up annually for 10 years, and was free of symptoms for the entire period. However, at the 10-year follow up interview, the patient reported a return of symptoms comparable to those experienced before the 10-year pain-free period. It was remarkable at this interview that an ultrasound probe with the beam in the “on” position placed with very light pressure on the brachial plexus in the interscalene area evoked the same symptoms as brachial plexus pressure, while the same probe with the same light pressure in the “off” position did not.

## LITERATURE REVIEW

### Anatomy of the thoracic outlet

The thoracic outlet lies between the shoulder and the neck, in the area between the clavicle and the thoracic cage. The thoracic outlet has three potential spaces.

**Figure 1:**
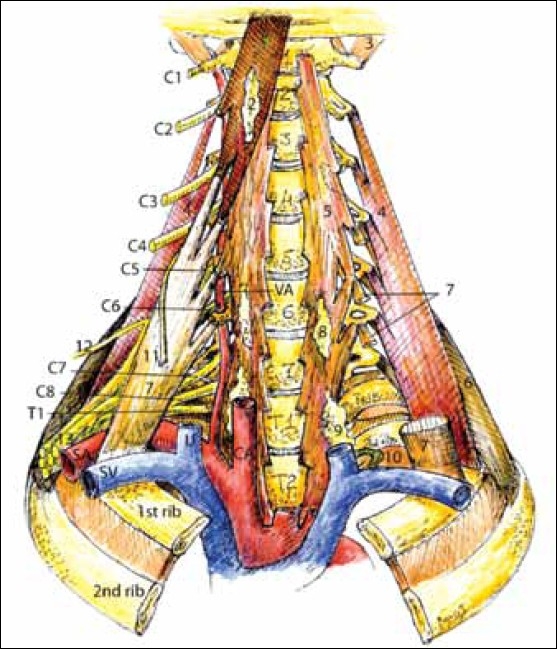
Deep cervical muscles, (1) Longus capitis muscle, (2) Superior sympathetic ganglion, (3) Rectus capitis muscle, (4) Middle scalene, (5) Longus colli, (6) Posterior scalene muscle, (7) Anterior scalene muscle, (8) Middle sympathetic ganglion, (9) Inferior sympathetic ganglion, (10) Thoracic duct, (11) Phrenic nerve, (12) Suprascapular nerve

The interscalene space: The interscalene space is bounded anteriorly by the anterior scalene muscle, posteriorly by the middle scalene muscle, and inferiorly by the portion of the clavicle between the insertions of these two muscles [Figure 1]. The anterior scalene muscle arises from the anterior tubercles of the transverse processes of the 3rd to 6th cervical vertebrae (C3-C6), and attaches to the scalene tubercle on the superior aspect of the first rib. The scalene tubercle separates the grooves formed by the subclavian artery laterally and the subclavian vein medially on the superior surface of the first rib. The middle scalene muscle arises from the posterior tubercles of the transverse processes of the 2nd to 7th cervical vertebrae (C2-C7), and inserts onto the posterior aspect of the first rib lateral to the groove of the subclavian artery and medial to the tubercle of the first rib. Enclosed in this triangle are the ventral rami of the 3rd to 5th cervical nerve roots (C3-C5) and the superior, middle, inferior trunks of the brachial plexus, and the subclavian artery. The superior (C5-C6) and middle (C7) trunk of the brachial plexus passes through the upper part of this space. The lower (C8-T1) trunk crosses the inferior part of the interscalene triangle behind the subclavian artery.[1] The subclavian vein passes medial to the anterior scalene muscle and lateral to the costoclavicular ligament. The scalenus minimus muscle, found to be associated with 30-50% of patients with NTOS,[2] originates from the transverse processes of C6 and C7 vertebrae, and inserts into the inner aspect of the first rib and pleura (Sibson’s fascia). Thus, over the top of the first rib, medial to lateral, are present the costoclavicular ligament, subclavian vein, anterior scalene muscle, subclavian artery, brachial plexus, and the middle scalene muscles. Anatomical variations in the scalene triangle have been associated with NTOS[3,4] [Figure 1].

The costoclavicular space: This is a triangular area bordered anteriorly by the medial aspect of the clavicle, posteromedially by the first rib, and posterolaterally by the upper border of the scapula. The costoclavicular ligament, as it arises from the medial aspect of the first rib cartilage, obliquely passes superolaterally to insert onto the undersurface of the medial aspect of the clavicle, forming a rough impression, the costal tuberosity (impression for costoclavicular ligament). The subclavius muscle originates from the junction of the first rib and its costal cartilage originates anterior to the costoclavicular ligament, and passes laterally and superiorly to be inserted into the subclavian groove on the undersurface of the clavicle situated lateral to the costal tuberosity. The subclavian vein courses posterior to the pectoralis minor muscle arches over the first rib and passes under the clavicle. It is situated lateral to the costoclavicular ligament and medial to the insertion of the anterior scalene muscle, thus providing a potential site for its compression as it traverses through the costoclavicular space.

The subpectoralis minor space: The triangular pectoralis minor muscle has its origin (base) from the 3^rd^ to 5^th^ ribs near their costal cartilages, and distally attaches (apex) to the medial border and superior surface of the coracoid process of the scapula. Along with the coracoid process, the pectoralis minor forms a “bridge,” under which neurovascular structures must pass to the arm; again, this presents a potential site for compression.

### Osteology

The superior border of the manubrium bounds the thoracic outlet anteriorly; laterally, it is bound by the inner surface of the first pair of ribs and their costal cartilages, and posteriorly by the first thoracic vertebra. The head of the first rib posteriorly articulates with the vertebral body, while the neck attaches to the transverse process. This bony circle, comprising the sternum, rib, and vertebra, forms the bony margins of the thoracic outlet. Anteriorly, the sternal end of the circle usually lies inferiorly in relation to the vertebral end, thus forming an anterior-inferior tilt to the outlet. The degree of this tilt varies among individuals, ranging from relatively flat to a significantly steep angle.

### Sono-anatomy

**Figure 2:**
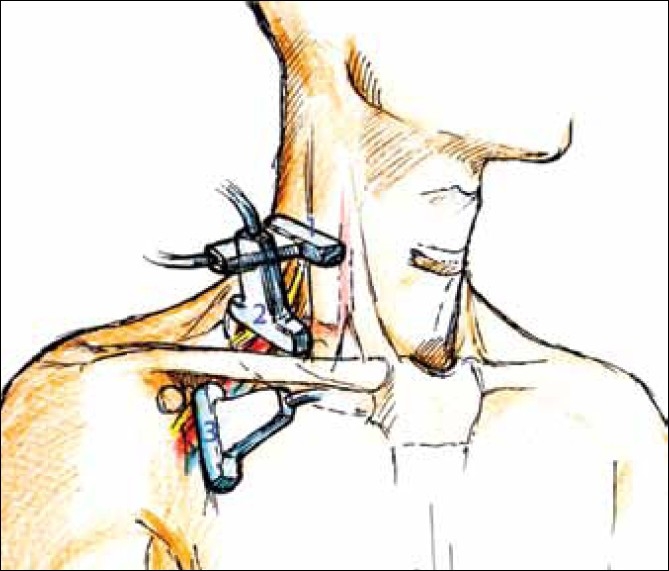
Probe position for Figure 3 – probe position 1, probe position for Figure 4 – probe position 2, probe position for Figure 5 – probe position 3

**Figure 3:**
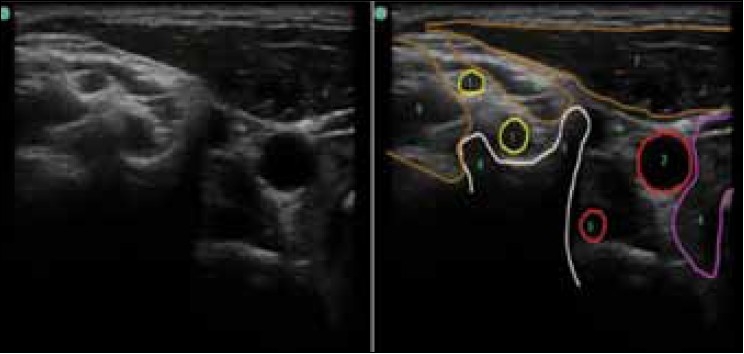
(1) Interscalene probe position at the level of C6 (position 1 in Figure 2) C5 and C6 nerve roots, (2) Carotid artery, (3) Vertebral artery, (4) Thyroid gland, (5) Anterior tubercle of the transverse process of C6, (6) Posterior tubercle of the transverse process of C6, (7) Sternoclydomastoid muscle, (8) Anterior scalene muscle, (9) Middle scalene muscle

**Figure 4:**
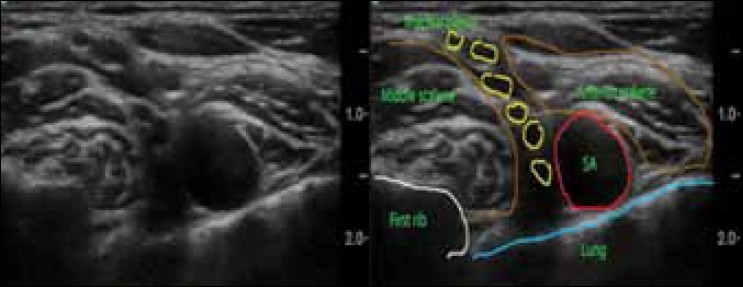
Supraclavicular interscalene probe position (position 2 in Figure 2) SA = Subclavian artery

**Figure 5:**
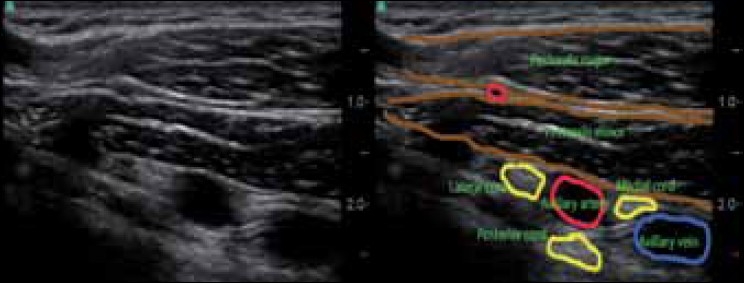
Infraclavicular probe position (position 3 in Figure 2)

Holding the ultrasound probe in positions 1, 2, and 3 as depicted in Figure 2, the sono-anatomy can be demonstrated as shown in Figure 3 (probe position 1), Figure 4 (probe position 2), and Figure 5 (probe position 3).

### Etiology

The thoracic outlet syndrome (TOS) is characterized by compression of neurovascular structures as they pass through the thoracic outlet. It affects approximately 8% of the population, with a female to male ratio of up to 4:1.[1] It is rarely seen in children, with a mean age of affected patients in the fourth decade.[1,5] Almost all cases of TOS (95-98%) affect the brachial plexus, with the other 2-5% affecting vascular structures, such as the subclavian artery and vein.[1,5] There are three separate spaces in the thoracic outlet where anatomical variation can potentially lead to TOS.[6] The first is the interscalene space, which is formed by the anterior scalene, middle scalene, and the first rib. The middle scalene, which is longer and thicker than the anterior scalene, has a variable insertion onto the first rib. Due to these variations, abnormal pressure can be exerted on the brachial plexus. A study noted that aberrant insertion of the middle scalene muscle can produce a “V” shape, which elevates the neurovascular structures within the interscalene space.[6] Additionally, a “U-shaped” sling (formed by the scalene muscles at the caudal aspect of the interscalene space) can occur if the insertion site of the middle scalene is closer to the anterior scalene insertion site, and can force the lower plexus upward. On occasion, the anterior and middle scalene muscles are fused above the plexus, which can put downward pressure on the upper plexus.[7] Additionally, first rib anomalies can lead to closure of the interscalene space. These include, but are not limited to, trauma, fracture callus, displaced fractures, subluxated clavicles, rib tumors, and hemangiomas, as well as fusion of the first rib with the second rib or cervical rib.[1,6–8] Cervical ribs are present in approximately 0.5-0.6% of the population, 50-80% of which are bilateral, and 10-20% produce symptoms; the female to male ratio is 2:1.[6] Cervical ribs and the fibromuscular bands connected to them are the cause of most neural compression. Roos and Poitevin have described nine and three arrangements of these fibrous bands, respectively.[9,10] The most common of these fibromuscular structures arises from a cervical rib and inserts at the intersection of the posterior portion of the first rib at its concavity and the insertion of the middle scalene muscle.[6] These arrangements can also be present in those without a cervical rib. A particularly long transverse process of C7 can be arranged the same way, with a fibrous band connecting it to the first rib.[5–7] The second anatomical space where TOS can occur is the costoclavicular space. This space is defined by the medial clavicle and subclavius muscle, the subclavian tendon, and costocoracoid ligament anteriorly, the first rib and insertion sites of the anterior and middle scalene muscles posteromedially, and the upper border or scapula posterolaterally. This space can be foreshortened by trauma, congenital anomalies, and changes to the muscles or tendons due to inflammation, spasm, or sprain.[6] Symptoms can present in patients with poor posture and sagging shoulders, or with a clavicle or first rib fracture with extensive callus formation.[1,6,7] They can be reproduced with shoulder abduction, which causes the scapula and coracoid to move downward, therefore putting inward pressure on the brachial plexus into the subclavius muscle and costocoracoid ligament.[5,6] Finally, the subpectoralis minor space is the third space where the brachial plexus can be compromised. Problems with this space are the least frequent in causing TOS. Hyper-abduction of the shoulder compresses the neurovascular bundle by the pectoralis minor below the coracoids.[5,6] Most patients with TOS have one of the aforementioned anatomic anomalies that predispose them to having symptoms. However, it is not until a physical injury to the neck or shoulder occurs that a patient will actually have symptoms. Many cadavers without a history of TOS-like symptoms have been found to have anatomic variations.[11] The physical injury is most often from a motor vehicle accident with a whiplash type injury.[5,7,11,12] Cumulative occupational trauma can also occur with any type of repetitive work that requires arms out ahead or overhead, such as using computers or working on assembly lines.[11] Poor posture with sagging shoulders is also a culprit.[1,6,7] Any high impact trauma to the shoulders or neck that leads to cervical muscle spasm can put additional traction on the anatomical variant structures in the thoracic outlet. These spasms lead to muscle edema and swelling, the combination of which compresses the brachial plexus, in turn, causing it to swell. Over time, the muscles become scarred and fibrotic, and they become fixed in a position that leads to constant compression of the brachial plexus.[5,7,11]

### Symptoms

When there is insufficient space for the brachial plexus as it travels from the neck to the axilla, symptoms such as pain, paresthesia, and weakness may develop. Mild symptoms can merely be positional, and resolve with repositioning of the arm. Two subtypes of neurogenic TOS exist.[5,13] Upper TOS involves the superior aspect of the brachial plexus (C5 through C7), with symptoms felt mostly in the arm and forearm, sparing the hand.[5,13] Additionally, the patient can experience neck pain on the affected side that radiates to the ear, face, and occiput, causing headaches.[13] The pain can also radiate across the rhomboids, clavicle, and trapezius/deltoid area. It can mimic a C5-C6 nerve root compression by a herniated nucleus pulposus.[5,13] Causes of upper TOS include hypertrophied scalene, passage of the brachial plexus through the anterior scalene, anterior pressure on the middle scalene by an elongated C7 transverse process, or the presence of a cervical rib.[13] Lower TOS involves the C8-T1 components of the brachial plexus.[5,13]. In these patients, the hand is affected, but the arm and forearm are spared. However, these patients can have neck and shoulder pain as well, with a variable intensity that can radiate down the medial brachial area of the arm into the forearm and hand. Paresthesias are usually felt in the 4th and 5^th^ digits.[5,13] Most patients with NTOS experience paresthesias in the fingers and sometimes the entire hand, usually in the median or ulnar distribution as described above. Also, if the arm is held overhead for a length of time, weakness may develop in the specific arm, and this fatiguing of the arm in overhead positions is often the presenting symptom. Furthermore, when carrying objects with the arm by the side (e.g. shopping bags) the arm may become numb and a loss of grip may occur. In more advanced cases, muscle atrophy can be seen, along with loss of fine motor movement.[13] Sleeplessness and irritability are likely. Most symptoms manifest after exercise, which helps to distinguish it from orthopedic injuries, which usually occur during exercise.[14] In addition, TOS is not limited to a specific dermatome, in comparison to a cervical disc problem which is usually dermatomal in nature.[13]

If patients’ symptoms are severe, they may have surgery to alleviate the anatomical problems. However, recurrence after surgery has an approximately 15-30% rate.[14] Excess scar tissue builds up at the surgical site, leading to re-entrapment of the brachial plexus with adhesions. Prevention consists of active and passive ROM exercises instituted promptly after surgery.[14] Symptoms of venous TOS include edema of the affected extremity, which happens acutely. The arm will also feel heavy, and collateral veins will appear more prominent. In more extreme cases, patients may develop thromboses due to cumulative damage to the intima during repetitive compression of the vein.[1,5,13] The destroyed intima will be a nidus for development of thrombus growth. In arterial TOS, patients may experience symptoms of arterial insufficiency, such as easy fatigability and paresthesias, as well as pain, coldness, and color changes in the hand and fingers of the affected side.[5,13] However, some patients may be asymptomatic until embolization develops. An embolus can form when the artery is repetitively compressed leading to a post-stenotic aneurysm. Thrombus can form inside the aneurysm and eventually embolize.[13]

### Diagnosis

There is no single diagnostic test that can confirm the presence of a NTOS. Variable presenting symptoms, complicated by multiple anatomical anomalies, present a diagnostic dilemma to the physician. Diagnosis is usually confirmed by a combination of a proper history and physical examination coupled with relevant radiological and electrophysiological studies.

#### Physical examination

A proper general physical examination should be supplemented with a focused examination of the neurological system. A focused examination may not only add credence to the diagnosis of NTOS, but may also point attention to other non-TOS etiologies. For example, an examination of the cervical spine could redirect suspicions to a cervical disc problem. It is essential that all muscle groups in the upper extremity be examined for degree of function and reflexes, in addition to a sensory examination of the relevant dermatomes. Neurogenic TOS is basically a compression of the brachial plexus, and thus, the C5-T1 nerves may be affected to a varying degree depending on the compression site.

Placing mild pressure with the thumb over the region of the brachial plexus in the supraclavicular fossa has been shown to reproduce symptoms in patients with NTOS, causing paresthesias down to the fingers.[13] A patient without NTOS would be asymptomatic. Other tests, like the Roos, hyperabduction and military tests[4] are commonly done, but while they may be of value for vascular TOS, these tests are not of any value for the diagnosis of NTOS.

The two most useful clinical tests often regarded as of great value for the diagnosis of NTOS are:

1. The “overhead stress test,” where the patient sits with both arms in the “90-90” position, the upper arms abducted to 90° and the elbows flexed to 90°. The hands are then opened and closed repeatedly. The symptoms of fatiguing and a burning sensation, often with paresthesia in the hand indicating a positive test.
2. The “downward pull test,” where the examiner exerts an axial traction force on the arm while holding the wrist of the patient. This may cause and simulate the symptoms of the patient as traction on the brachial plexus is exerted.

#### Anterior scalene block

An anterior scalene muscle block, where local anesthetic agent is injected directly into this muscle, may paralyze the muscle, and reduce the pressure exerted by it on the brachial plexus. This may aid in the diagnosis if spasm of this muscle is the primary etiology. However, inadvertent blockade of the somatic and sympathetic nerves may occur, and this could mask a complex regional pain syndrome (CRPS) if the sympathetic nerves were blocked, or it may mask true shoulder pathology if the brachial plexus were blocked.

Electrophysiological guidance has been shown to assist in needle tip positioning. In a study by Jordan *et al*, of the 38 patients who underwent surgical decompression of the thoracic outlet, 30 of 32 (94%) had a positive outcome following an anterior scalene block and a good outcome following surgery, compared to 3 of 6 (50%) who underwent surgery and had good results in spite of a negative block.[15] The results of these blocks appear to correlate with surgical outcomes.

#### Radiological investigations

Radiological investigations may aid in confirming etiology related to a cervical disc or differentiating between a vascular and neurogenic TOS.

Cervical spine X-rays: Cervical spine films should be evaluated for the presence of degenerative cervical spine disease, cervical ribs, and other structural abnormalities of the first rib and clavicle.

Arteriography and venography: Arteriography may be indicated if an aneurysm is suspected; however, in most patients, an arterial TOS is confirmed by symptoms and physical examination. It may be useful if surgical correction of arterial TOS is under consideration. Venography may help in diagnosing a venous TOS.

CT and MRI imaging: These imaging modalities can primarily detect cervical disc disease, scalene muscle anomalies, and brachial plexus compression. They may help diagnose a Pancoast tumor or metastatic involvement of the brachial plexus.

### Electrodiagnostic studies

Electrodiagnostic studies fall under two categories: electro-myography (EMG) and nerve conduction studies (NCS). EMG studies have been found useful in the diagnosis of neurogenic TOS, but may not be sensitive enough in patients with a milder form of the disease.[16,17] Motor NCS can test the brachial plexus motor component at the root or cord level. Any decrease in amplitude of response is suggestive of axonal loss. Delayed conduction may implicate a demyelinating disease.[17] Differentiation between preganglionic and postganglionic pathology may be possible with sensory NCS.[18,19] Somatosensory-evoked potential studies have been utilized to diagnose neurogenic TOS,[20,21] but may be nonspecific.[18,22] More recently, medial antebrachial cutaneous (MAC) NCS have been measured to aid in the diagnoses of neurogenic TOS.[23] MAC NCS measure sensory function in the lower trunk of the brachial plexus. Abnormal findings in MAC NCS were found in patients with normal EMG and NCS, thereby aiding the diagnoses of milder forms of neurogenic TOS.[24]

### Treatment

TOS presents challenges for the physician in both diagnosis and management. Once TOS has been determined, the first step is conservative therapy.

#### Conservative therapy

Most authors suggest a trial of 3 to 6 months of conservative therapy prior to undergoing surgical intervention.[25] Kenny, *et al* recommends 4 to 6 months of conservative therapy based on a prospective study evaluating patients who underwent a specific neck and shoulder exercise regimen.[26] A survey of practitioners found that all attempted conservative therapy for 2-12 months, with an average of 6 months.[27]

There are various treatments that can be utilized for a patient with TOS, and most benefit from a combination of medications in conjunction with physical and occupational therapy. Usually, a physical therapy regimen begins with patient education. Therapists work with the patients to correct workspace and home ergonomics.[28] Improved posture during sitting, standing, and sleeping often reduces edema and tension, and thus ameliorates symptoms.[28] Therapists also concentrate on stretching exercises. Wehbe describes a series of nerve gliding exercises that can be employed to safely relieve tension on the nerves of the brachial plexus during arm and neck movements.[29] If applicable, weight loss or reducing breast size can often decrease tension on the brachial plexus. In these cases, some studies suggest that women change their bra to one with a wider shoulder strap.[28] In the past, physical therapy has employed strength training, weight lifting, and neck traction. Although such modalities are helpful for other neck and shoulder injuries, caution should be taken in cases of TOS.[25] Conservative interventions include using a TENS (transcutaneous electrical nerve stimulator) unit. Massage therapy performed by a therapist knowledgeable about the TOS can be helpful as well. Many patients benefit from formal relaxation sessions and deep breathing. Biofeedback has been useful, particularly a system called the *Feldenkrais method*.[30] This system of educating the patient to change physical behaviors can be incorporated into their physical therapy and rehabilitation programs, and has proven useful.

Along with a regimen of physical therapy, patients often respond to muscle relaxants and anti-inflammatory medications. Together, these treatments help most patients with NTOS; however, no prospective study involving multiple treatment modalities has been performed to identify which patients will successfully avoid the need for surgery.[25]

#### Vascular thoracic outlet syndrome treatment

Venous TOS, also called *Paget-Schroetter syndrome or effort thrombosis*, can usually be attributed to both an underlying anatomical abnormality and concomitant repetitive arm raising exercises, such as swimming or throwing a ball.[31] There is therefore a preexisting narrowing of the subclavian vein that results in an acute and/or chronic thrombosis. It is most common on the patient’s dominant side. Systemic thrombolysis is a mainstay of treatment, although endovascular thrombolysis has now become more widely available and utilized for the acute onset of symptoms. Regardless of the initial treatment chosen, all patients should be placed on long term anticoagulation.[31]

In a prospective study conducted over a 21-year period, all the patients presenting with acute subclavian vein thrombosis were treated with thrombolysis, and underwent immediate first rib removal, scalenectomy, removal of the subclavius muscle, and vein patch of the stenotic subclavian vein segment.[32] In the 17 patients who had delayed surgery in this series after their initial thrombolysis, 70% were inoperable secondary to fibrosis and proximal occlusion of the neurovascular structures. All of the inoperable patients had some degree of arm disability. For patients with chronic venous TOS who are candidates for surgical intervention, the preferred method is with the use of a venous patch.[33] Rethrombosis of the subclavian vein was associated with use of stents after thrombolysis and angioplasty, and age younger than 28.[34] Similar to venous TOS, an underlying bony abnormality is usually the cause of the arterial TOS.[35] This is most commonly a cervical rib, but can also be an anomalous first rib or both a cervical rib and anomalous first rib. Acute presentation almost always requires urgent surgical intervention. Revascularization is possible, but surgical resection of the cervical rib or first rib is necessary to prevent further arterial lesions and potential emboli.[36] In the Cormier study, 35 of 39 patients followed after cervical rib or anomalous first rib resection had no symptoms. Those patients had a mean follow up of 5 years, 8 months. Patients with arterial vascular outlet syndrome will often have distal emboli. In one study, all patients with the arterial form of the syndrome had distal emboli requiring revascularization.[37] Although occlusion secondary to thrombosis and stenosis produces the symptoms and acute threat of limb ischemia in this disease, angiogram often shows aneurysms with or without thrombus of the subclavian artery.[35] Therefore, surgical treatment in addition to bony resection should include resection and reconstruction of the affected artery segment.

#### Botulinum toxin

Botox is a neurotoxin originating from *Clostridium botulinum*, a gram-positive anaerobic bacteria. It inhibits calcium-dependent acetylcholine release from presynaptic nerve endings at the neuromuscular endplate,[38] and, thus, generates chemodenervation of the muscles injected, in this case the middle and anterior scalene muscles. Botox (Botulinum toxin type A) was first used in TOS in 1939 for diagnosis.[39] A more contemporary study by Jordan showed that not only is Botox injection into scalene muscles helpful for diagnosis, a positive response also increases the likelihood of success after surgical intervention.[15] Recently, Botox has been used more for treating symptoms of the syndrome. The pain of NTOS can be linked to interscalene muscle hemorrhage and swelling, particularly after an acute precipitating injury, such as an automobile accident.[40] This injury is usually related to hyperextension of the shoulder. Scar tissue and fibrosis subsequently form, as will be evident histologically, which exerts pressure on the brachial plexus and the subclavian artery, thus effectively narrowing the scalene triangle.[41] Injecting Botox into the scalene muscles, therefore, can relax those muscles and relieve symptoms. Using fluoroscopy and electromyography, Jordan compared interscalene muscle injections with Botox and lidocaine plus steroids.[42] Fourteen of the 22 patients (64%) who received Botox reported at least a 50% decrease in their symptoms compared to 18% of patients who received lidocaine plus steroids. All patients who reported relief of their symptoms had that benefit for at least 1 month after the injection, with a mean of 88 days in the Botox group. No side effects were reported in the Botox group, except for mild and transient dysphagia in 2 of the 22 patients in this group.

No prospective study has yet been undertaken to prove the efficacy of using Botox injections to treat TOS. However, there is one ongoing randomized, double-blinded and placebo-controlled study at the University of British Columbia that will evaluate pain, paresthesias, and quality of life changes.[43] The practitioners in this study use EMG guidance, and all patients in both arms of the study undergo physical therapy with stretching and strengthening exercises. In addition to its use in NTOS, Botox injections have been used for the nonacute arterial form of TOS. A case report by Danielson and Odderson showed a significant difference in subclavian artery flow rates when the affected arm is abducted with external rotation before and after the Botox injection.[44] Using ultrasound guidance for these injections has gained favor. One retrospective study of scalene muscle Botox injections compared those done under ultrasound guidance (n=77) versus blocks under fluoroscopy (n=168). Both with electromyography showed a 91% and 81% improvement, respectively, in TOS symptoms.[45] The authors also did not find any statistically significant differences in complication rates between the two, although patients receiving injections under fluoroscopy are subjected to more radiation.

### Surgery for neurogenic thoracic outlet syndrome

Surgical intervention for NTOS is a mainstay of therapy; however, given that it carries a significant risk of complications, surgery should not be considered until the patient has[2] undergone a thorough diagnostic evaluation,[3] has had several months of conservative therapy, and[4] demonstrates that there is a disability involved with the condition.[25] Multiple surgical modalities exist, and depend largely on the patient’s anatomy and the surgeon’s preference. Surgeons can perform a cervical rib resection and a first rib resection, using either a cervical or transaxillary approach and anterior scalenotomy or scalenectomy, or a combination of these. Lord performed claviculectomies beginning in 1953, but this procedure has largely been abandoned as other, less disfiguring surgeries have gained popularity.[46]

Surgeons have reported similar results using different techniques for surgical decompression of the brachial plexus. In one study, patients with NTOS underwent an anterior and middle scalenectomy with or without a first rib resection.[47] The authors used a supraclavicular approach for the scalenectomy and the first rib resection. Patients with cervical ribs were managed differently, as the first rib was removed, and they were not used in the study. No difference in success rates was noted between the two groups. However, patients who had rib-sparing surgery developed less pleural injuries (40% vs. 59%) and had shorter hospital stays. The authors cite the duration of symptoms less than 2 years prior to surgery as the only factor predictive of success.

The finding, that there is no significant difference in success rates between the two surgical approaches, has been echoed by several other studies. In a review article, Mackinnon cites a 90% success rate in multiple studies immediately postoperatively, and an average of 70% success rate if the patients were followed for 5-10 years after surgery.[48] Another literature review by Sanders showed no significant difference in surgical success whether by transaxillary first rib resection, supraclavicular first rib resection with scalenectomy, or supraclavicular first rib resection without scalenectomy.[25] The success rate overall after 5 years of follow-up, according to Sanders, is approximately 68%.[49] The findings suggest that first rib resection is, therefore, not necessary, as it does not improve success rates, but increases morbidity and recovery time. Sanders further suggests that a first rib resection is effective in decreasing symptoms because the surgery itself is also a scalenotomy by removing the insertion site of the anterior and middle scalene muscles.[49] When a cervical rib is present, Sanders does perform a rib resection during the first surgical intervention. Mattson also prefers a supraclavicular approach to anterior and middle scalenectomy.[50] Loh employs a supraclavicular approach as well, citing that 94% of his patients had improvement postoperatively, and 74% had complete relief of symptoms.[51] In contrast to Sanders’ supraclavicular approach to the first rib resection, when the resection is necessary, Atasoy promotes a combined method involving a transaxillary approach to the first rib resection, followed immediately by transcervical scalenectomy.[52] In one study, he showed that 95% of 102 patients displayed improvement in their symptoms after surgery.[53] Wehbe uses a supraclavicular approach to the anterior scalenectomy and partial middle scalenectomy, but stresses the importance of epineurectomy for full neurolysis of the brachial plexus.[54] His approach employs careful dissection starting at the C5 root to the T1 root and anteriorly to all three trunks of the plexus. Of the 52 patients in this study, only 2% of the patients with severe pain continued to have severe pain in the postoperative period. They determined a 100% success rate in that all the patients in the study would elect to have the surgery again. The authors in this study do contemplate the theoretical possibility of devascularization of the nerves, although they have found no clinical evidence of this result. Authors cite various rates of complications, depending on their approach, but also on their experience with the surgery. Brachial plexus injury is usually a product of traction on the nerve roots. Sanders cites plexus injury in 1% of his patients, and states that injury can be reduced by changes in positioning during surgery, specifically by flexing the neck toward the operative side.[49] Phrenic nerve injury is usually transient, and develops in 6-12% of patients.[25] This injury tends to be more common with the supraclavicular approach.[49] One percent of patients have a long thoracic nerve injury resulting from transaction of a branch of the nerve during dissection of the middle scalene muscle.[49] This injury is also usually transient because other branches of the nerve remain intact. Horner’s syndrome has a risk of 0.5-1%, and is almost always transient and more common with a supraclavicular approach.[49] During left-sided surgery, injury to the thoracic duct is a concern; it occurs in 2-3% of those cases, and is more prevalent with the supraclavicular approach. Pneumothorax develops in 2-15% of patients and is more common when surgeons perform a first rib or cervical rib resection compared to rib-sparing surgery.[53] Other complications include seromas, hemothorax, and injury to the subclavian vein and artery. Surgeons often refer to decreased rates of complications when using an approach by which they feel exposure is increased and with which they have experience, and therefore confidence. Overall, there is no significant difference in success or complication rates that can guide which surgical approach is superior.[55,56] No prospective data exist comparing transaxillary vs. supraclavicular approaches, nor is there prospective data comparing results with surgery and no surgery. Additionally, there have been no comparisons of chemoneurolysis with Botox and surgical neurolysis. With long term follow-up data showing an average of 65-70% success after surgical intervention, there is much room for improvement in the surgical technique and diagnostic stratification.[56,57]

## CONCLUSIONS

NTOS is a complex syndrome that requires thorough knowledge of the anatomy and anatomical variants. Because this syndrome involves the brachial plexus, it is advisable not to offer brachial plexus blockade to patients undergoing shoulder or other upper limb surgical procedures until the pathology is clear and brachial plexus block is clearly indicated. Ultrasound may prove to be an asset in the diagnosis and treatment of NTOS.
